# Merkel cell carcinoma metastatic to the testis: report of a rare diagnosis and review of the literature

**DOI:** 10.4322/acr.2020.198

**Published:** 2020-12-08

**Authors:** Davide Gigliano, João Lobo, Paula Lopes, Ivo Julião, Francisco Lobo, Rosa Azevedo, Rui Henrique, Ângelo Rodrigues

**Affiliations:** 1 Instituto Português de Oncologia do Porto, Serviço de Anatomia Patológica, Porto, Portugal; 2 Research Center of Portuguese Oncology Institute of Porto (GEBC CI-IPOP), Porto Comprehensive Cancer Center (P.CCC), Cancer Biology and Epigenetics Group, Porto, Portugal; 3 Instituto de Ciências Biomédicas Abel Salazar, Universidade do Porto (ICBAS-UP), Departamento de Patologia e Imunologia Molecular, Porto, Portugal; 4 Instituto Português de Oncologia do Porto, Serviço de Oncologia Médica, Porto, Portugal; 5 Instituto Português de Oncologia do Porto, Serviço de Urologia, Porto, Portugal

**Keywords:** Carcinoma, Merkel cell, Neoplasm Metastasis, Neuroendocrine Tumors, Testis

## Abstract

Merkel cell carcinoma is an aggressive malignancy that frequently recurs/disseminates, but metastases to the genitourinary tract are rare. Only eight cases of Merkel cell carcinoma metastatic to the testis are reported. We describe the ninth case of this event and provide a review of the literature. A 58-year-old man diagnosed with Merkel cell carcinoma of the wrist, presented, 37 months later, a recurrence in the form of a testicular metastasis. The tumor consisted of a monotonous proliferation of small, blue, round cells, with immunoexpression of neuroendocrine markers and the typical dot-like paranuclear immunostaining for cytokeratin 20, in the absence of immunostaining for cytokeratin 7. The patient is alive with no evidence of disease. Clinicians should be aware of the possibility of metastatic dissemination to the testis since genital examination/imaging is not part of routine follow-up for these patients, but timely orchiectomy may be curative.

## INTRODUCTION

Merkel cell carcinoma (MCC) is an uncommon, and aggressive cutaneous neuroendocrine neoplasm, mostly located in the head and neck region (40-50% of the cases) with a high rate of local recurrence or distant metastasis (80%) within 2-3 years after diagnosis.^1^ ^,^ ^2^

Occult metastatic dissemination is already present at diagnosis in 30% of patients, mostly to lymph nodes. Distant metastatic sites include the liver, bone, lung, brain, adrenal gland, and skin.^1^ Metastases to the genitourinary tract are less common.

To the best of our knowledge, only eight cases of Merkel cell carcinoma metastatic to the testis were reported in the English literature. Herein, we report the ninth case and review the literature.

### Case Report

**Figure 1:**
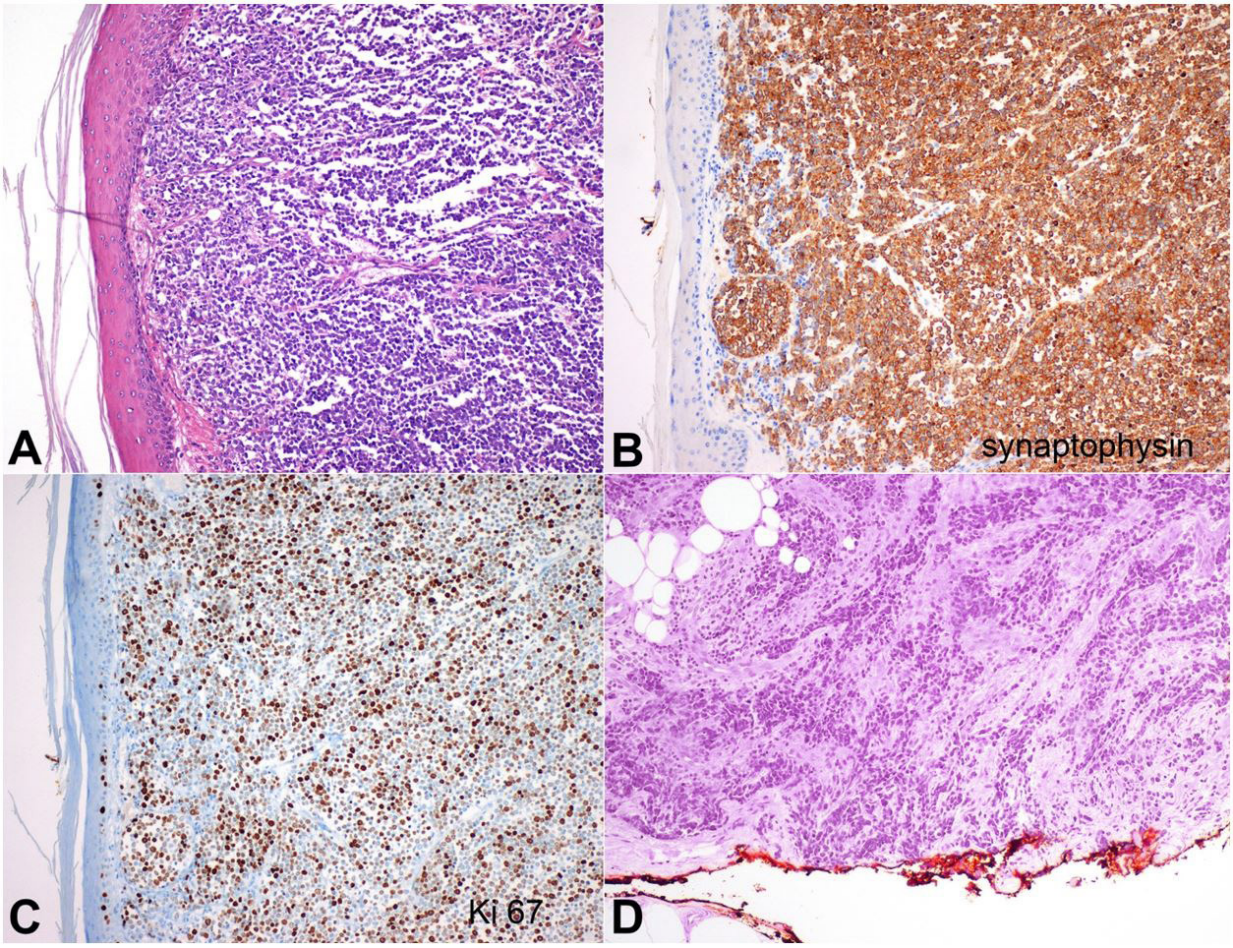
Photomicrograph of the skin. **A –** The lesion had a solid growth pattern (H&E, 100X); **B –** It was diffusely positive for synaptophysin (100X); **C –** Ki67 index was high (100X); **D –** The tumor was present in the surgical margin (notice tumor cells present at the red-inked margin) (H&E 200X).

A 58-year-old man was diagnosed with MCC of the left wrist and underwent local excision in a peripheral center. The tumor was composed of dense and diffusely infiltrative blue cells centered in the dermis (Figure 1A), diffusely positive for Synaptophysin (Figure 1B), CK20 (in the dot-like pattern) and the Ki67 index was high (Figure 1C). As the resection margins were not free of tumor (Figure 1D), the patient was referred to the Oncologic Institute.

At the first appointment, he presented a remaining ulcerating lesion on the wrist and no further complaints. He had a history of hypertension, adenoma of the adrenal gland, obesity, hyperuricemia, and colonic diverticulosis. The computed tomography scan showed a large homolateral axillary mass. He underwent wide local excision of the wrist tumor and the axillary lymph node dissection. As the histopathological examination showed evidence of metastatic disease (Figure 22B), and invasion of adipose and muscular tissues, chemotherapy (four courses of cisplatin, 150.4mg in 1000mL NaCl 0.9%, and etoposide, 188mg in 500mL NaCl 0.9%) and radiotherapy (60Gy in 30 fractions to the left wrist and 60Gy in 30 fractions to the left axilla) were included in the patient’s therapeutic regimen.

**Figure 2:**
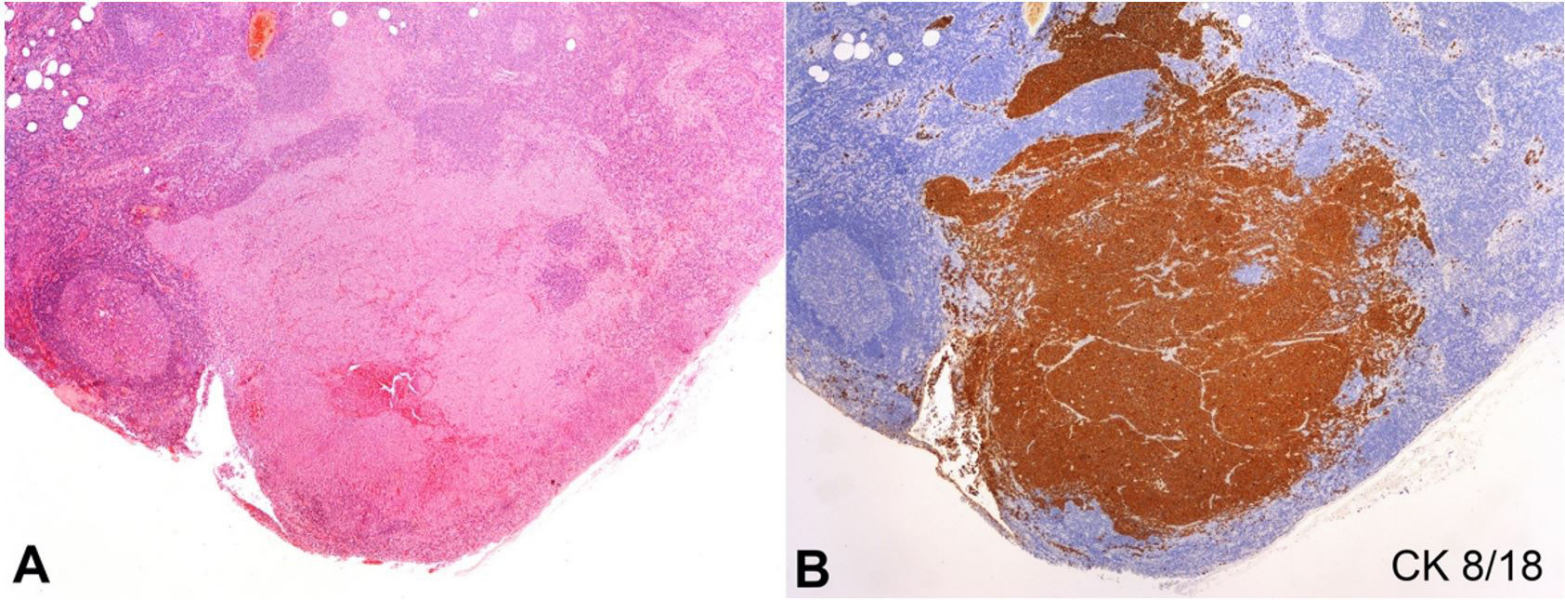
Photomicrograph of the lymph node showing in **A –** metastatic infiltration (HE, 200X); **B –** positive reaction for CK 8/18 (40X).

**Figure 3:**
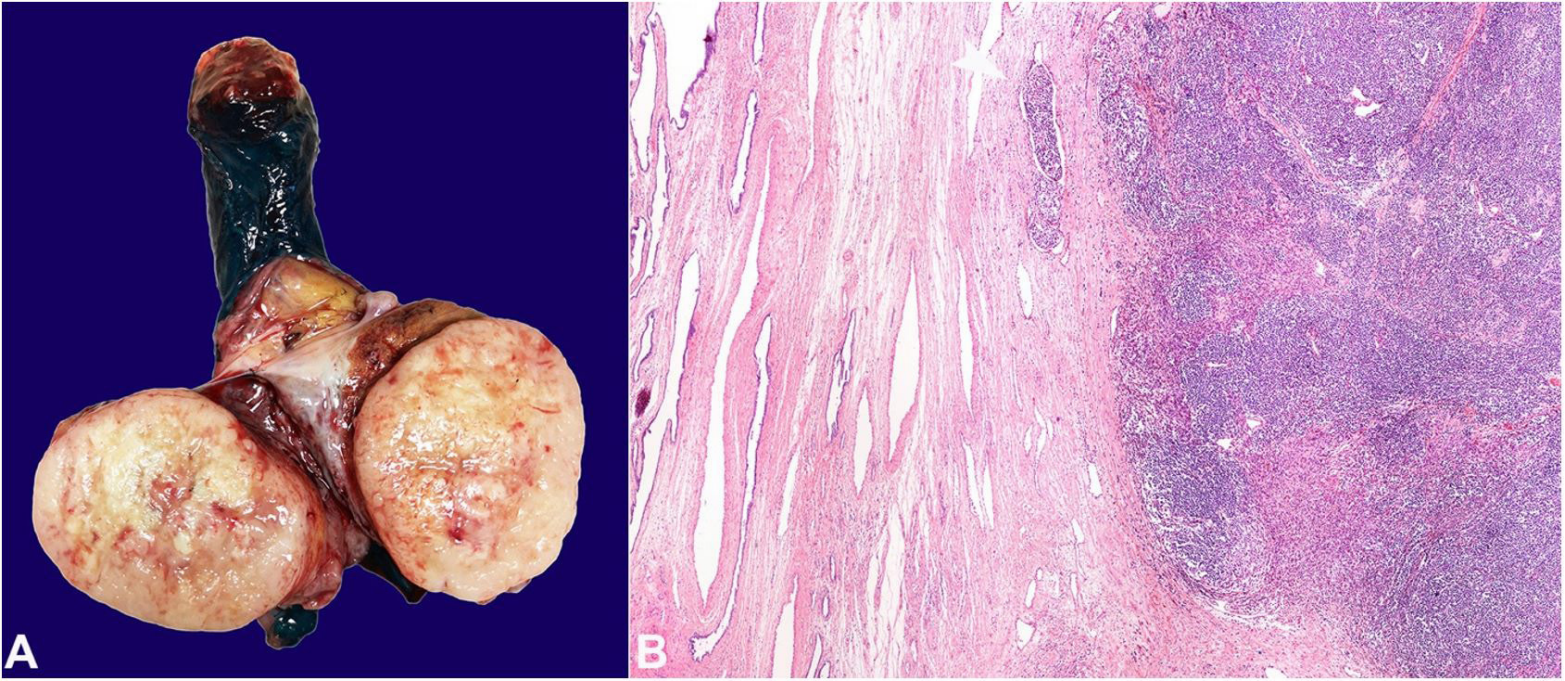
Macroscopic aspect (A) and histological overview (B) of the Merkel cell carcinoma within the testis. **A –** The lesion was solid, tan, with foci of hemorrhage and necrosis, and replaced almost the entire testicular parenchyma, sparing the mediastinum testis; **B –** The lesion was well demarcated and confined to the testicular parenchyma, sparing the rete and mediastinum testis. Vascular invasion was easily identifiable (white arrow).

Thirty-seven months after MCC initial diagnosis, the patient complained of recent-onset scrotal edema. The physical examination disclosed a solitary, indurated nodule within the left testis. The scrotal ultrasound revealed a heterogeneous mass. The laboratory workup was unremarkable, including normal serum alpha-fetoprotein (AFP), human chorionic gonadotropin subunit beta (β-HCG), and lactate dehydrogenase (LDH). The patient underwent left radical orchiectomy. The macroscopic examination revealed a 60×49×40mm white to tan, ovoid, solid mass, almost completely replacing the testicular parenchyma (Figure 3A). Centrally, areas of necrosis were depicted. On histological examination, the mass corresponded to a highly cellular, monotonous proliferation of small, blue, round cells. On low power field examination, the tumor exhibited a solid growth pattern, extensively infiltrating throughout the seminiferous tubules, being confined to the testis (Figure 3B). Lymphovascular invasion was easily identified.

**Figure 4:**
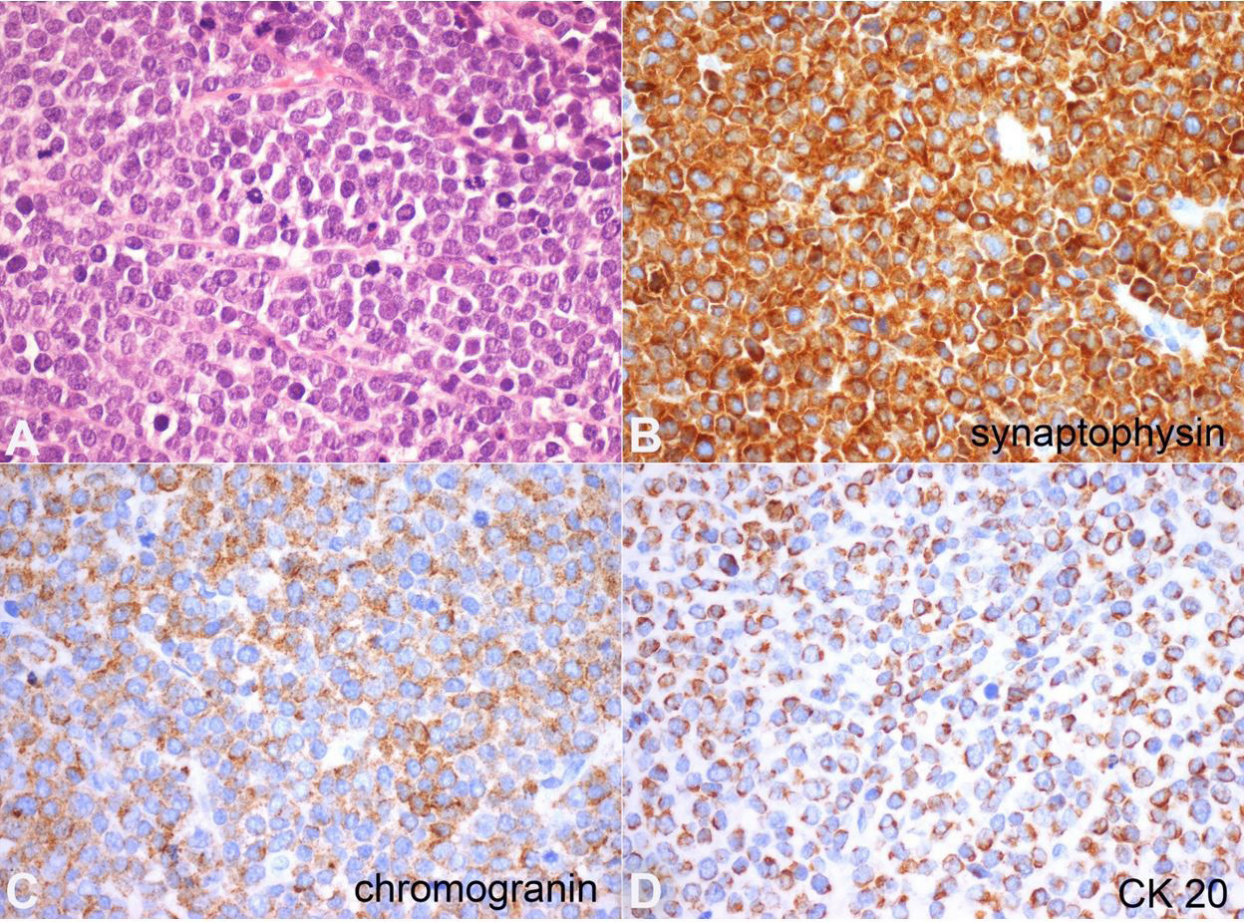
Photomicrographs of the tumor - Histological and immunohistochemistry studies of the testis’ Merkel cell carcinoma. **A –** The neoplasm had a solid, somewhat organoid growth pattern, and was composed of tumor cells with scant eosinophilic cytoplasm, hyperchromatic nuclei with coarsely granular chromatin and visible nucleoli. Numerous mitotic figures were evident (H&E,400X); **B –** Tumor cells disclosed strong and diffuse immunoexpression of synaptophysin (400X); **C –** multifocal and robust reaction to chromogranin (400X); **D –** strong and diffuse reaction to CK20 (400X).

On the higher magnification, the tumor cells were often arranged in an organoid and trabecular fashion, displaying a scant eosinophilic cytoplasm, hyperchromatic enlarged nuclei with coarsely granular chromatin, visible nucleoli and brisk mitotic activity (Figure 4A). The immunohistochemistry panel (see Table 1) revealed a diffuse and robust immunoexpression of synaptophysin (Figure 4B), chromogranin (Figure 4C) and CD56; dot-like para nuclear immunostaining for cytokeratin 20 (Figure 4D) and cytokeratins 8/18; and lack of immunoexpression for cytokeratin 7, CD45, PLAP, OCT 3/4, TTF-1, CDX2 and PS100 (Table 1). A diagnosis of metastatic involvement by MCC was made.

**Table 1:** Immunohistochemistry methods.

**Antibody**	**Clone**	**Vendor**	**Dilution**	**Detection system**	**Automatic platform**
**Synaptophysin**	DAK-SYNAP	DAKO	1/150	BPRD kit	LB III
**Chromogranin**	DAK-A3	DAKO	1/200	BPRD kit	LB III
**CD56**	CD564	LEICA	1/100	BPRD kit	LB III
**Cytokeratin 20**	Ks20.8	LEICA	1/250	BPRD kit	LB III
**Cytokeratin 7**	OV-TL 12/30	LEICA	1/150	BPRD kit	LB III
**CAM 5.2**	5D3	LEICA	1/100	BPRD kit	LB III
**CD45**	2B11 & PD7/26	DAKO	1/300	BPRD kit	LB III
**PLAP**	8A9	DAKO	1/100	BPRD kit	LB III
**OCT 3/4**	C-10	MASTER DIAGNÓSTICA	Prediluted	BPRD kit	LB III
**TTF-1**	8G7G3/1	CELL MARQUE	1/200	BPRD kit	LB III
**PS100**	Polyclonal	DAKO	1/3000	BPRD kit	LB III
**Ki67**	MIB-1	DAKO	1/200	UUDD kit	Ventana

BPRD kit: Bond Polymer Refine Detection Kit; LB III: Leica Bond III; UUDD kit: Ultraview Universal DAB Detection Kit.

The patient is currently under surveillance. He is alive with no other signs of disease, 47 months after the initial diagnosis of MCC.

## DISCUSSION

MCC is an overall rare neoplasm (0.1 to 1.6 per 100.000 people per year, depending on the region of the globe), which is more prevalent in males and the elderly (median age at diagnosis of 75-80 years).^1^ Incidence has been rising in the past years (an age-adjusted annual increase of 8%, greater than the one observed for melanoma), and the mortality rate is high (33%), also higher than that of melanoma.^2^ MCC has been linked to exposure to ultraviolet light and also to Merkel-cell polyomavirus, which recently has been used as rationale for treatment with immunotherapies, namely pembrolizumab. The five-year survival rate of patients with distant metastases is 25%, what means a guarded prognosis. The increase in life expectancy, and the substantial increase in the incidence of MCC, impose the need for accurate detection of such metastatic disease. Therefore, the screening for the most common, and the unusual metastatic sites should take part in the clinicians’ approach. Indeed, the metastases to the genitourinary tract are not among the most common sites. Specifically, to date, only eight cases of MCC metastatic to the testis were reported in the literature (Table 2), our report is the ninth case.^3^ ^-^ ^9^ Metastasis to the genital tract is accessible for curative surgical excision. It may be overlooked since the external genitalia is not routinely examined in the MCC surveillance. Neither included in the imaging workup.^4^ All reported cases were, indeed, detected in the presence of symptomatic enlargement of the testis, except in one case in which metastatic dissemination was depicted in both testes by somatostatin receptor scintigraphy.

**Table 2:** Reported cases of Merkel cell carcinoma metastatic to the testis

Author	Clinical feature	Age (y)	Side	Size (cm)	Primary MCC	Rx for primary MCC	Rx of testicular lesion	Time to metas (m)	Metas sites	Order of events	Follow-up
Present case	Testicular swelling	61	Left	6	Left wrist	Resection + LN dissection (positive) + RT + CT	O	37	LN	PR→O	ANED (47 months after primary tumor diagnosis)
Mweempwa et al.^3^	Painless testicular swelling	66	Bilateral metach (3 M interval)	7; 3	U	N/A	O + CT	N/A	None	O →CT	ANED (26 months after second O)
Whitman et al.^4^	Testicular mass	70	Right	2.4	Right gluteus	Resection + SLN biopsy (positive) + LN dissection (negative)	O	15	None	PR →O	ANED (6 months after O)
Tummala et al.^5^	Testicular mass	54	Right	N/A	Left forearm	Resection + LN dissection (positive) + RT + CT	O	2	None	PR →O	ANED (36 months after O)
Schwindl et al.^6^	Painful testicular swelling	70	Right	N/A	Right forearm	Resection + RT + LN dissection (positive)	O	7	LN	PR →O→Sg + CT	DFD (14 months after the primary tumor diagnosis)
Gleason et al.^7^	Painless testicular mass	53	Left	2.4	Right gluteus	CT + RT	O	22	Shoulder, lung, mediastinum, LN	PR → meta→ RT → O → metas → palliative RT	DFD (12 months after O)
Rufini et al.^8^	No testicular symptoms (detected by SRS)	38	Bilateral synch	N/A	U	N/A (received CT)	None	2	LN	Metastasis biopsy → CT → SRS (positive in testes)	DFD (8 months after primary tumor diagnosis)
Ro et al.^9^	Painless enlargement	73	Right	6	Upper lip	Resection + RT + iridium implants	O + CT	18	LN, subcutaneous, liver	PR → meta	DFD (6 months after testicular metastasis)
	Testicular swelling	47	Bilateral metach (2 m interval)	N/A	Left elbow	Resection + RT + CT	Bilateral O	12	No	PR → O	ANED (12 months after first testicular metastasis)

ANED: alive with no evidence of disease; CT: chemotherapy; DFD: died from disease; LN: lymph node; M: months; meta: metastasis; MCC: Merkel cell carcinoma; metach: metachronous; NA: not available; O: orchiectomy; PR: primary tumor resection; RT: radiotherapy; Rx: treatment; Sg: surgery; SRS: somatostatin receptor scintigraphy; Synch: synchronous; U: unknown.

The median age at diagnosis of the MCC testicular metastases is 60 years (range: 38-73 years), similar to our patient (61 years old), but below the median age of 75 years described for diagnosis of primary MCC. MCC is intimately related to immunosuppression, which favors earlier clinical presentation, besides a poorer prognosis.^10^ Our patient was obese, had hepatic steatosis and hyperuricemia, features consistent with chronic alcohol consumption, which may express a sort of immune system deficiency. It has also been hypothesized that metastatic dissemination of MCC to the testis may reflect the effect of the hematotesticular barrier, which prevents accessibility of chemotherapy agents, failing to eradicate minimal residual testicular disease.^3^ ^,^ ^5^

The time from primary tumor diagnosis to clinical detection of metastasis within the testis varied between 2 to 22 months (median 12 months), in consonance with the data on recurrences elsewhere, which is the first 2-3 years.^1^ ^,^ ^2^ Our patient presented the testicular metastasis after 37 months of the primary diagnosis, constituting the largest time interval reported to date in this specific setting.

Four out of the eight reported patients (50%) died of the disease. In the present case, considering the presence of a large metastatic axillary mass with surrounding tissue invasion, a combination of both adjuvant chemotherapy and radiotherapy to the primary tumor site and axilla was pursued, as considered in the guidelines.^11^ Despite the recurrence in the testis (treated by orchiectomy alone), a good outcome was achieved (the patient is alive with no evidence of disease 47 months after the primary tumor diagnosis).

The differential diagnosis of MCC metastatic to the testis can be challenging. The Pathologist should have a high index of suspicion since some patterns may mimic primary testicular tumors.^12^ The immunohistochemistry studies aid to clinch the precise diagnosis. More than 95% of testicular masses correspond to germ cell tumors, which are heterogeneous and may simulate several entities;^13^ however, most afflict young-adult patients. The overall histological pattern, elder patients (over 60 years), and negativity for PLAP and OCT3/4 discarded this hypothesis. Metastases to the testis/paratesticular area are overall rare (with the reported incidence varying from 0.06% to 3.6%), and most frequently arise from the prostate.^12^ In our case, the known clinical history of a previous MCC with metastases to the lymph-nodes was determinant and raised suspicion. In the reported cases, most MCC metastases to the testis were unilateral (like in our case), which should not refrain from considering a metastasis. MCC should be differentiated from other entities with possible similar histological and cytological features, namely small-cell lung cancer (small-cell) lymphomas and anaplastic small-cell melanomas. Negativity for CD45, TTF-1, and PS100 aid ruling out these entities. Expression of neuroendocrine markers (synaptophysin, chromogranin, and CD56) and, importantly, the typical dot-like pattern of CK20 immunoexpression (in the absence of CK7 immunoexpression) further confirm the diagnosis. Of note, since very rarely small-cell carcinoma of the lung may show immunoexpression of CK20, and also very rarely MCC may be negative for CK20 and positive for TTF-1, it is wise to use a panel of antibodies, combined with clinical information, instead of relying on a single antibody and risk a misdiagnosis.^1^ Also, when lymphoblastic lymphoma is a concern, it is wise to have in mind that TdT expression occurs in MCC.^14^

To conclude, we report the ninth case of metastatic dissemination of MCC to the testis. A high level of clinical suspicion and accurate histopathological assessment, including immunohistochemistry studies, are necessary for the precise diagnosis. Clinicians should be aware of this unusual pattern of dissemination since orchiectomy can be curative.
